# GigaGauss solenoidal magnetic field inside bubbles excited in under-dense plasma

**DOI:** 10.1038/srep36139

**Published:** 2016-10-31

**Authors:** Zs. Lécz, I. V. Konoplev, A. Seryi, A. Andreev

**Affiliations:** 1 ELI-ALPS, Dugonics Square 13, 6720 Szeged, Hungary; 2 John Adams Institute for Accelerator Science and Physics Department, University of Oxford, Keble Road, Oxford OX1 3RH, UK; 3 Max-Born Institute, Berlin, Germany; 4 Sankt Petersburg State University, St. Petersburg, Russia

## Abstract

This paper proposes a novel and effective method for generating GigaGauss level, solenoidal quasi-static magnetic fields in under-dense plasma using screw-shaped high intensity laser pulses. This method produces large solenoidal fields that move with the driving laser pulse and are collinear with the accelerated electrons. This is in contrast with already known techniques which rely on interactions with over-dense or solid targets and generates radial or toroidal magnetic field localized at the stationary target. The solenoidal field is quasi-stationary in the reference frame of the laser pulse and can be used for guiding electron beams. It can also provide synchrotron radiation beam emittance cooling for laser-plasma accelerated electron and positron beams, opening up novel opportunities for designs of the light sources, free electron lasers, and high energy colliders based on laser plasma acceleration.

Magnetic fields are one of the fundamental entities which influence nature on all scales. They shapes planets and stars^1^; electron beams in laser-plasma particle accelerators are guided and focused by magnetic fields^2^,^3^ and they are used to understand natural phenomena under extreme conditions^4^. Magnetic fields are also used to stimulate coherent x-ray radiation from charged particle beams^5^. The capability to generate high strength magnetic field is essential for many projects and research communities (see for example MegaGauss International conferences). The MegaTesla magnetic fields are expected to exist inside neutron stars^6^ and can be used to understand matter behavior under extreme conditions^7^. Fields of up to 1 kT strength are currently being generated using explosive magnetic generators^8^ or high-current single shot targets driven by pulsed power generators^9^, which are used to generate high intensity X-ray fluxes. Such machines are capable of generating magnetic fields of high strength (140 T)^10^ and there are predictions of possible generation up to 600 T in some cases^11^. The single shot techniques are well developed and allows generating of fields around 1 kT. Alternatively, using solenoids in non destructive machines, the maximum field strengths are reported to be 100 T at Los Alamos National Laboratory^12^. Reaching a GigaGauss strength magnetic fields of levels seems to be impossible as the electron currents required to drive such fields and **J** × **B** self induced forces will destroy any currently available material.

Developments of laser technology stimulated exploration of methods for generating large magnetic fields from laser pulses directly or via interaction with plasma or solid targets. The close method to conventional techniques uses pulsed solenoids and a kilotesla magnetic field was generated by pulsed laser in a capacitor-coil target configuration^13^. Predictions of large toroidal magnetic field generated by laser pulse in near-critical plasma were made in ref. 14 and experimental observations reported in refs 15, 16 and 17 demonstrated production of megagauss magnetic fields in laser interactions with solid targets. In all these cases, the field is generated in a stationary space volume; the target is either solid or near-critical plasma and direction of the field is radial or toroidal, i.e. not solenoidal and not quasi-static in co-moving electron beam frame.

More recently, the generation of a longitudinal magnetic field was explored^18^ using laser pulses carrying orbital angular momentum (OAM) created by polarization of the laser pulse^19^,^20^ or a hollow screw-like intense LaguerreGaussian laser pulse^21^. These techniques are closer to those discussed in this paper but the method suggested is capable of producing significantly higher solenoidal fields. It is shown that this new method is different from the inverse Faraday effect (IFE) considered in the case of circularly polarized laser pulses^22^,^23^.

The generation of high amplitude solenoidal fields described in this paper is based on interaction of screw-shaped laser pulse^19^ interacting with an under-dense plasma. This interaction creates a GigaGauss magnetic field within a volume of 0.1–10*s μ*m transverse dimensions, depending on the plasma density and pulse spirality. This moves with the laser pulse and thus is suitable for effective interaction with the accelerated electron or positron beams. The generation of the solenoidal field can also be achieved by using charged particle (electron) beams and will also be considered in following studies.

The generation of a screw-shaped laser pulse is interesting in its own right^20^. It is assumed that the shaped pulse can be generated via compression and focusing along a single rotating plane of circular polarized beam or shaping it with relativistic electrons^24^,^25^ and amplifying the generated higher harmonics^20^. An alternative and probably more efficient method uses wavefront rotation. This has been successfully applied for intense short pulses in the attosecond lighthouse effect^26^ (see Supplementary Material).

In this paper, the bubble solenoid (short solenoid) and steady solenoid (long solenoid) operating regimes will be discussed. In the first regime, the solenoidal magnetic field is generated by the electron currents moving around a plasma bubble shell in a similar manner to the solenoid. The highest field is observed at the end of the bubble where electrons trajectories are collapsing creating high strength longitudinal magnetic field. In this case, the field’s strength is adiabatically changing from the center of the bubble to the end, reaching the highest amplitude. The magnetic field direction coincides with the direction of laser propagation with the volume (few cubic micrometers) having the highest field, defined by the step of the laser spiral. The second regime is realized when a plasma bubble is not formed under a set of conditions (discussed below) and the solenoid currents are not limited to the dimensions of the plasma wavelength. In this case the field can be sustained for longer distances, until the pulse energy is depleted but the field amplitude achieved in this regime will be order of magnitude less, i.e. 10 *s* kT.

The paper has a description of the computation model in the second section and includes a discussion of the application of the bubble solenoid regime to laser plasma acceleration of electron or positron beams, demonstrating the fast synchrtotron radiation cooling of the emittance of the accelerated beams. The third section discusses different regimes. In the conclusions, the results are summarized and outline future work and possible impacts. The capability to generate such fields will allow significant progress in non-destructive, reproducible studies of phenomena at fields strength which are not yet accessible in the laboratories. In this work, we focus on the novel opportunities opened for design of laser-acceleration based devices – light sources, free electron lasers and high energy colliders.

## Electron blow-out and collapse

The laser-plasma (LP) interaction is one of the most dynamic research areas which is driven by numerous problems including design of compact light sources and particle accelerators. The generation of MegaTesla (MT) magnetic fields is another challenge which can be resolved using LP interaction with parameters being different from conventional studies^27^ and in general may not be optimal for other applications e.g., LP acceleration of electrons.

The average ponderomotive force of a pulse, which is roportional to the gradient of the intensity envelope function, acts on the electrons^28^,^29^ under conditions where the plasma wavelength, *λ*_*p*_, is much larger than the laser wavelength, *λ*_*L*_, where *λ*_*p*_ = 2*πc*/*ω*_*p*_ and *ω*_*p*_ = (*n*_0_*e*^2^/(*γm*_*e*_*ε*_0_))^1/2^ and *n*_0_ is the unperturbed plasma density, *γ* is the relativistic factor and *m*_*e*_ the mass of electron.

This envelope model is widely used for the investigations of laser wake field acceleration (LWFA) in low density plasmas^30^. This appropriate and valid “short-cut” minimizes the spatial resolution of numerical modeling and leads to reduction of calculation time. In this work the effects of laser pulse shape and its angular momentum on the bubble formation and magnetic field generation is studied using 3D Particle in Cell (PIC) code VSim, which has a self-consistent light-frame envelope model implemented based on the method from ref. 30.

**Figure 1:**
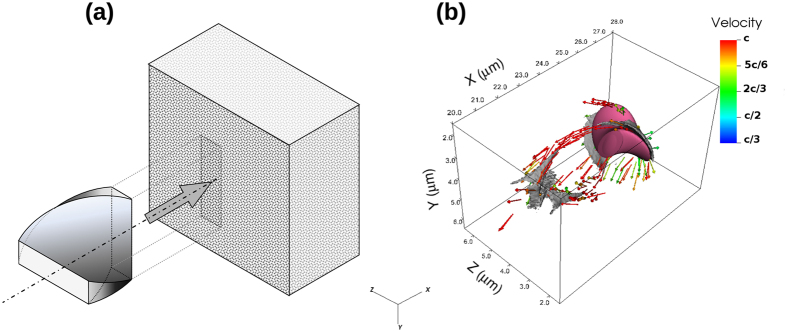
(**a**) Schematic of the laser pulse moving into the plasma (grey box) with momentary projection of the pulse front onto the box. (**b**) Illustrates the isosurface of pulse intensity (purple) at the value of 3 × 10^20^ W/cm^2^ and electron density (gray) with 10^26^ m^−3^. The arrows represent the velocity vectors of electrons seen from the moving frame of the laser pulse and the color code shows their magnitude.

The exact analytical description of the laser pulse is presented in the Supplementary Material. The envelope function of the laser pulse shape is illustrated in Fig. 1a, and is similar to a drill bit propagating along x-coordinate. Figure 1b shows the electron dynamics whilst the pulse moves through the plasma. The intensity distribution of the pulse is Gaussian-like, but is modulated helically, providing the desired spiral shape. The main difference in plasma dynamics between the standard Gaussian and the spiral-shaped beam propagation through the plasma is the azimuthal non-uniformity of electron density i.e. the appearance of azimuthal current along the bubble surface.

The electrons expelled by the laser pulse will move along spiral trajectories on the surface of the generated bubble (azimuthal electron current), which in turn induces strong axial magnetic field with the maximum value in the back of the bubble. The spiral shaped laser pulse gives a twist to the electrons which can be seen at the right side of Fig. 1b (in the vicinity of the pulse). Two electric current channels are formed as the electrons travel to the back side of the bubble. They approach each other and finally pass within a distance less than laser spot diameter. These two electron currents are in the opposite direction, can not merge and thus repel each other, limiting the magnetic field generated at the merging point. This motion of the dense electron bunches is shown in Fig. 1b. To minimize the calculation time and for a better illustration of the electron currents, the frame co-moving with the laser pulse is used. Throughout this paper, the laser wavelength has been adjusted so that the conditions *λ*_*L*_ ≪ *λ*_*sp*_, where *λ*_*sp*_ is the spiral step, and *n*_0_ < *n*_*cr*_, where 
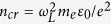
, are fulfilled.

In the example shown in Fig. 1b, the peak intensity is *I*_0_ = 1.6 × 10^21^ W/cm^2^; the pulse width is 1.8 *μ*m (FWHM); the pulse length is equal to the spiral step, *λ*_*sp*_ = 1.8 *μ*m; and electron density is typical of fully ionized C-H gas density, *n*_0_ = 0.62 × 10^−3^*n*_*cr*_ = 7 × 10^25^ m^−3^. In order to use the envelope model, the plasma has to be strongly underdense, i.e. *n*_0_/*n*_*cr*_ ≪ 1, to ignore the pulse energy depletion during the short time scale considered here. The pulse evolution during propagation in underdense plasma is not trivial^31^. The exact pulse and field evolution over a longer time is beyond the scope of this current work and it will be studied in the near future.

Although the plasma is dilute, the pulse shape can be strongly modified at the edges of the radial Gaussian profile, where the edge of the helical intensity modulation is trimmed by the repelled electrons. This reduces pulse spirality and results in a much smoother quasi-Gaussian beam. The spiral step can, however, be decreased due to pulse steepening^27^ at the front of the laser pulse which change the field distribution at the back of the bubble. All these effects make the interaction extremely complicated, therefore this study is limited to a narrow parameter-range and time window where the generated B-field is quasi-static. Later in this paper, there are examples where the pulse evolution can not be neglected even on this short time scale.

**Figure 2:**
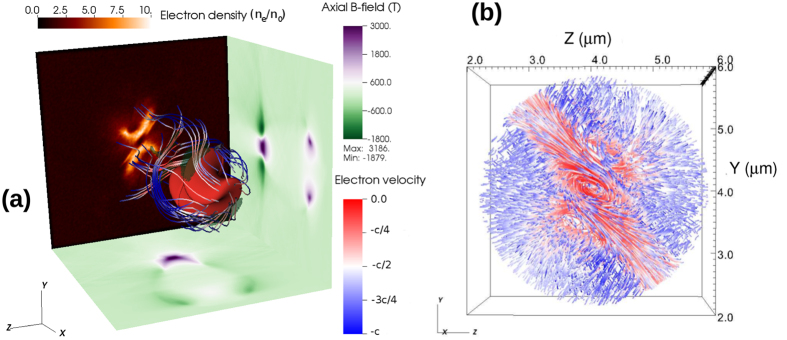
(**a**) A three-dimensional view of the electron streamlines. The cross section of axial magnetic field and charge density of electrons are shown on the visualization box faces. The transparent green isosurface illustrates the regions where the electric current is the highest. (**b**) Zoomed image of the streamlines in the back of the bubble.

The complex dynamics of electrons fluxes and the mechanism of the field generation is more elucidated in Fig. 2a, which shows the electron streamlines flowing around the bubble in three dimensions. The cross sections of the longitudinal magnetic field, taken along the axis of the propagation, are shown on the lateral sides of the simulation box. The back side presents the transversal cross section of the electron density at the tail of the bubble. A more detailed image of the structure of the electric currents is shown in Fig. 2b. This shows that a part of the electron tracks simply pass through and leave the highly concentrated region while some electrons will be captured by the electromagnetic (EM) potential and start to move on a spiral path. A small portion of the electrons moves outward and will spiral in the opposite direction (the negative B-field seen in the *xy* plane of the left picture).

The electron trajectory and bubble shape strongly depends on the balance between the kinetic energy of electrons gained in the force field of the laser pulse and on the EM potential generated by the bubble. If the latter is high enough, electrons can be captured and accelerated while moving in the strong magnetic field. This electron dynamics can be beneficial for improving an accelerated electron beam emittance in laser plasma accelerators. The internal structure of the longitudinal magnetic field depends also on the number of captured electrons and the efficiency of B-field generation can decrease if the number of accelerated electrons is too high.

## Magnetic field structure and scaling with parameters

### The bubble solenoid

**Figure 3:**
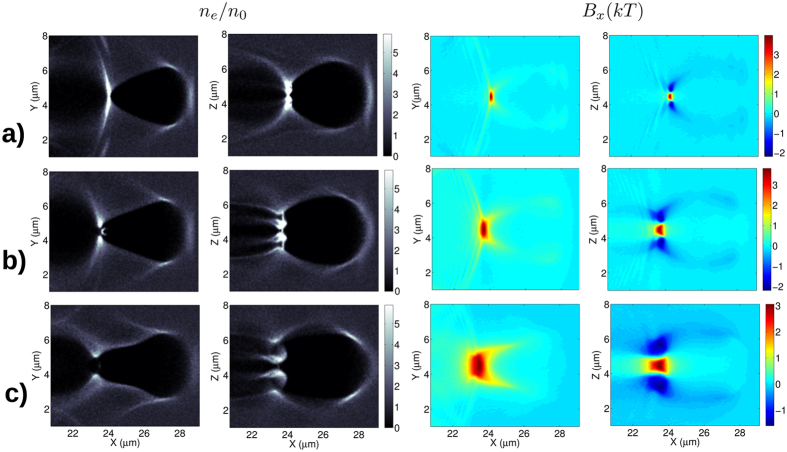
Cross sections of electron density normalized to the initial density in the two orthogonal transversal planes and the corresponding magnetic fields in the same planes for rows: (***a***) *λ*_*sp*_ = 0.9 *μ*m; (***b***) 1.8 *μ*m; and (***c***) 3.6 *μ*m. In all cases, *n*_0_ = 0.62 × 10^−3^*n*_*cr*_.

**Figure 4:**
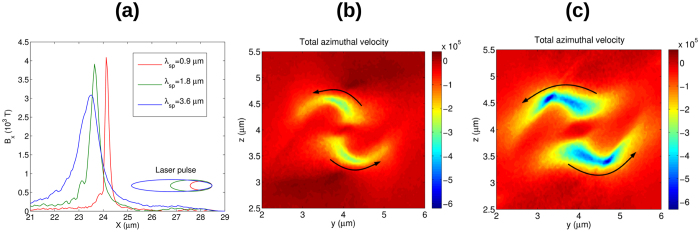
(**a**) Longitudinal magnetic field along the axis of propagation for different spiral steps of the laser pulse presented in Fig. 3(b,c) show the total azimuthal velocity (∑*vφ*/*c*) distribution in the transverse plane at the back of the bubble for *λ*_*sp*_ = 0.9 *μ*m and *λ*_*sp*_ = 3.6 *μ*m, respectively. The negative velocity (blue colors) indicates clockwise rotation while small positive or near zero velocities (red colors) indicates anti-clock rotation.

Focus now shifts to the case where the collapsing electrons can escape the attracting field of the bubble and do not disturb the generated magnetic field. Here the dependency of the magnetic field on plasma density, laser pulse spiral step, intensity and wavelength will be investigated. All these parameters define the electron currents surrounding the bubble which drive the GGs (GigaGauss) level magnetic field. Simulations were performed in order to check the dependence on the spiral step with the results presented in Fig. 3. In all cases, the pulse length is equal to the pulse spiral step, meaning a 180 degrees rotation of the intensity profile, but, due to the Gaussian longitudinal profile, the effective rotation is roughly 90 degrees (Fig. 1). It can be clearly seen that the magnetic field profile becomes more solenoidal in the *xz* plane as the pulse length becomes comparable with the bubble size. The peak axial magnetic field is more localized in the case of short *λ*_*sp*_ but is more elongated in the case of larger *λ*_*sp*_. The normalized electron density distribution is shown in the left panels of Fig. 3 and it is clear that the charge densities in *xy* and *xz* planes are different as a result of “assymmetric” laser pulse. Higher compression is visible for the smaller spiral step resulting in an observed higher peak of magnetic field, Fig. 4.

The shape of the magnetic field distribution and the actual peak value depend on the details of complex electron’s trajectories and on the level of compression acquired in the tail of the bubble. However, the self-induced repulsive forces can result in destruction of the currents and thus limits the amplitude of generated magnetic field. According to Fig. 3 the transversal size of the bubble does not depend on the spiral step, thus the compressed electron density scales as *n*_*e*_ = (*λ*_*p*_/*λ*_*sp*_)*n*_0_. The second thing to be noticed is that the magnetic field is generated by finite current sheets with thickness approximately *λ*_*sp*_/2. Applying Ampere’s law to this geometry, the magnetic field is found to be *B* = *μ*_0_*j*_0_*λ*_*sp*_, where *j*_0_ = *en*_*e*_*v*_*φ*_ and *v*_*φ*_ is the azimuthal velocity. Using this expression for the compressed electron density results in *B* = *pμ*_0_*en*_0_*λ*_*p*_*v*_*φ*_/2, where *p* < 1 indicates the percentage of electrons that contribute to the transverse current. Using *λ*_*p*_ ≈ 8 *μ*m results an estimated value of *B* ≈ *p* ⋅ 16 kT for the B-field. This is close to measured values (see Fig. 4) assuming that only quarter of the electrons are contributing to the transverse current.

Figure 4a shows that the peak magnetic field only weakly depends on the spiral step (or pulse length in this setup) but through the expression of ponderomotive force it can be shown that


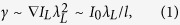


where *l* = *λ*_*sp*_/*λ*_*L*_. On the other hand the bubble length (or half plasma wavelength) is proportional to 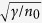, thus


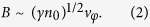


This scaling suggests that the axial magnetic field shown in Fig. 4a should be two times lower for 4 times longer spiral step. However, the peak values are much close to each other, which can be attributed to the larger azimuthal momentum acquired with larger *λ*_*sp*_. The comparison of azimuthal velocity distribution at the back of the bubble is shown in Fig. 4b,c for *λ*_*sp*_ = 0.9 *μ*m and *λ*_*sp*_ = 3.6 *μ*m, respectively. The number of rotating electrons is the same in both cases, but a longer spiral step result in a two times greater azimuthal velocity, which compensates for the reduced gamma factor.

The magnetic field generation with screw-shaped pulses differs from the IFE, where the B-field is generated inside of the laser pulse without formation of bubble or wakefield. IFE depends on the field polarization not on the pulse shape. Moreover the derived IFE scaling predicts much weaker field strengths^23^: 
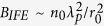
, where *r*_0_ ≫ *λ*_*p*_ is the plasma channel radius.

It is straightforward to expect higher magnetic field inside larger volumes by increasing the laser intensity (the energy available to drive the current) and plasma density (current available to generate the field). In the following, it is proven that near GGs level magnetic fields can be experimentally generated in the laboratory environment using laser intensities obtainable with current (or foreseen) technology. Table 1 shows the parameters used in the next simulations. The first parameter-set (Sim1) has been used in the simulations presented above. The bubble size is defined by laser spot size (*W*_*L*_ = 2*σ*_1_), laser intensity and plasma density and in order to have similar bubble structure as before, the laser spot size is between one third and half of the bubble length.

**Figure 5:**
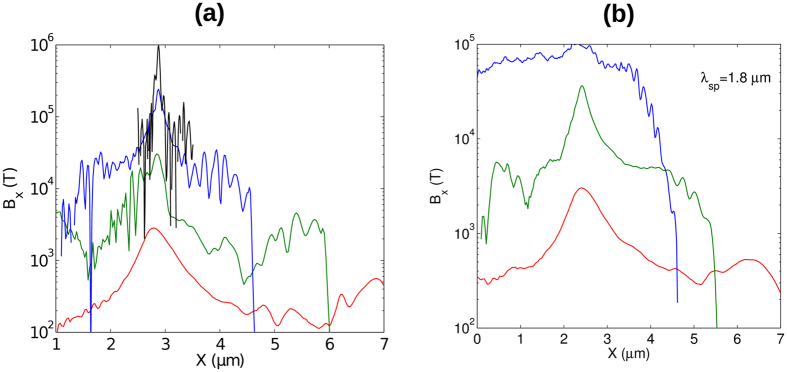
Longitudinal magnetic field along the axis of propagation for parameters shown in: (**a**) Table 1 and (**b**) for two times larger spiral step. The red, green, blue and black lines corresponds to simulations 1, 2, 3, 4 respectively. The color code is the same in both pictures.

The axial magnetic fields measured for the first four parameter-sets are shown in Fig. 5a,b. The previously derived expression predicts a scaling *B* ~ (*γn*_0_)^1/2^ in the strongly relativistic case, i.e. *v*_*φ*_ ≈ *c*. This is close to the observed scaling. In the case of larger spiral step (Fig. 5b), the third simulation did not give the expected result because the pulse was longer than the bubble and the magnetic field generation enters a different regime - this will be discussed later. In the case of Sim4, the plasma density is near the solid density which forms a very small bubble and requires a reduction in the laser wavelength and pulse size. Although the resulting magnetic field reaches the MT level, the required laser parameters are not currently obtainable with available laser technology. However, it is possible to scale the parameters such that the resulting B-field remains the same. For this, the following condition has to be fulfilled:


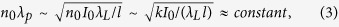


where *k* = *n*_0_/*n*_*cr*_. The following constrain is required for the same bubble shape:


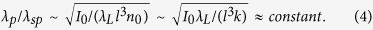


Unfortunately by increasing the laser wavelength up to 0.8 *μ*m, the plasma becomes overdense which results in quick depletion, by absorption, or even reflection of the laser pulse and no axial B-field is generated. At this stage it can be speculated that the issue can be resolved if an electron beam with angular momentum is used to drive the currents capable of generating MT magnetic fields.

**Figure 6:**
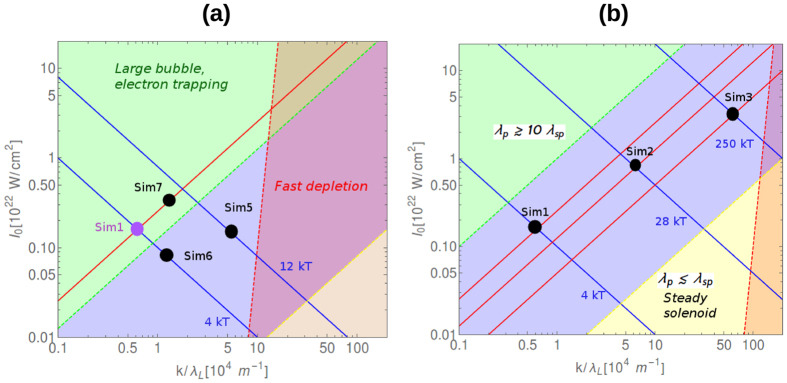
Parameter-map of the bubble solenoid generation, where the region of different regimes are also indicated, for two laser wavelengths: (**a**) *λ*_*L*_ = 800 nm and (**b**) *λ*_*L*_ = 100 nm. The blue and red lines (Eqs (3 and 4)) show the parameters required to generate the same magnetic field amplitude and the same field distribution, respectively. The blue area shows the parameter interval discussed in this section: the bubble solenoid regime. The red dashed line corresponds to *k* = *n*_0_/*n*_*cr*_ = 0.1, behind which the laser pulse depletion affects the magnetic field generation and the model should be revisited.

From previously presented expressions it follows that either the bubble shape or magnetic field amplitude are changed by modifying the laser or plasma parameters. In the above simulations, the dimensionless pulse length was *l* = 9 and if it is kept the same, the laser wavelength and intensity can be tuned, within well-defined limits, to obtain the same magnetic field strength. In Fig. 6 shows the iso-value curves of max(*B*_*x*_) and *λ*_*p*_/*λ*_*sp*_ which indicate the parameter plane scaling for constant pulse length *l*. The simulations discussed and presented in Table 1 are shown on these planes of parameters. The blue area indicates the parameter space where the bubble regime can be observed. The green area shows the regime of strong electron trapping which is not favorable for generation of large magnetic fields, the yellow area corresponds to the regime of steady solenoid (presented in the next section) and the red area is the area where the plasma density is large and pulse energy depletion is no longer negligible. The yellow and green dashed lines are not exact curves and transitions from green to blue and from red to yellow are always expected due to energy depletion. It is clearly visible that the laser wavelength strongly influences the results and pushes the blue area towards lower intensity and density regions, which in turn means weaker magnetic field generation. High intensity and short wavelength are required for the generation of high B-field in static bubble solenoid.

**Figure 7:**
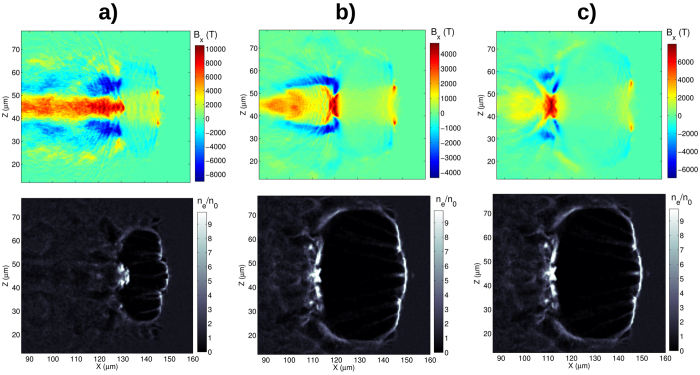
Longitudinal magnetic generated in the upper row and normalized electron density in the lower row observed for the parameter sets shown in Table 1: (**a**) Sim5, (**b**) Sim6 and (**c**) Sim7.

In order to prove the validity of the scalings, the magnetic field and electron density cross sections from simulation 5, 6, 7 are shown in Fig. 7, where the laser wavelength is 8 times longer than in Sim1. By comparing Fig. 7a (*k* = 0.04) with Fig. 3a (*k* = 0.62 × 10^−3^) it can be seen that the field amplitude increases by factor of 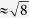 and the relative bubble size, *λ*_*p*_/*λ*_*sp*_, decreases by the same factor, ≈2.8. A large number of captured electrons decreases the electrostatic potential inside of the bubble leading to its expansion. The laser intensity and plasma density can be changed so that roughly the same magnetic field (Sim6) and larger *λ*_*p*_/*λ*_*sp*_ ratio (Fig. 7b) is obtained. Similar bubble shapes can also be obtained by increasing the laser intensity and decreasing the plasma density (Sim7 in Fig. 7c).

**Figure 8:**
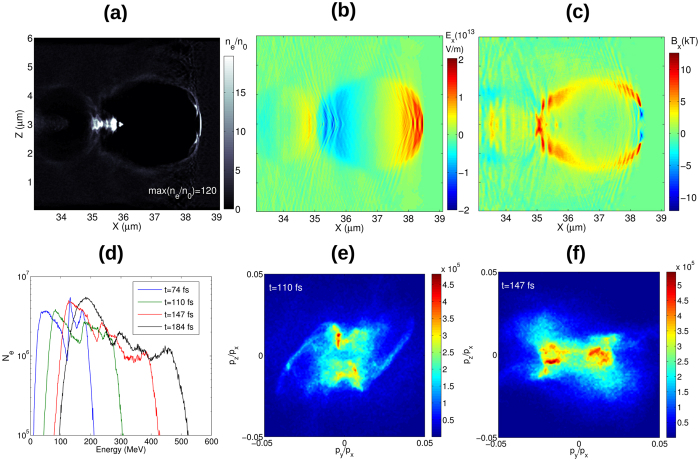
(**a**) A snapshot of electron density, (**b**) longitudinal electric field and (**c**) axial magnetic field in the case of Sim2 at *t* = 110 fs. **(d)** Electron energy and **(e,f)** momentum distributions for different time instances.

Plasma waves produced in the under-dense plasmas are capable to accelerate electrons to GeV energy. In the case of screw-like-shaped laser pulse the longitudinal accelerating field is combined with strong longitudinal magnetic field which is pointing in the same direction. This gives a set of new features to this accelerating scheme. The B-field produced in the tail of the bubble results in a very large number of electrons being accumulated in the accelerated beam with dramatically improved emittance. In Fig. 8 the electron energy has reached 500 MeV after only 60 *μ*m of propagation. This high energy is due to the high plasma density and laser intensity used in the simulation. The energy spectrum and angular distribution of electrons is shown in Fig. 8d–f. Two electron bunches are observable in the density distribution (8a), which appear also in the momentum distribution. An efficient acceleration and multiple beam bunching is visible, even in this preliminary studies. The angular spread of the electrons decreases as their energy increases and they probably will be unified into a single dense and energetic electron bunch if the acceleration is sufficiently long. The energy spread of electrons is relatively large but the angular spread of the individual bunches is less then 1 mrad. The large energy spread can be attributed to pulse energy depletion and to the large charge density of the electron beam which modifies the bubble fields (Fig. 8b,c) reducing the bunching and acceleration effectiveness.

These electrons are compressed due to the strong magnetic field and rotate along the propagation axis (Fig. 8e,f). This electron beam dynamics could be very interesting for generating high intensity synchrotron radiation (SR) at <nm wavelength scale, predominantly in the forward direction. The spatial resolution of this simulation method is not high enough to resolve these wavelengths (and the spiral motion of electrons) and this radiation can not be investigated at this stage of the numerical modeling. This will be studied in the future using extended PIC codes^32^. It is anticipated that, due to electron spiral motion in magnetic field, strong energy loss from the transversal motion will occur due to intense SR. The SR losses per unit length are^33^:


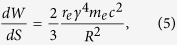


where *r*_*e*_ is the classical electron radius and *R* is the radius of curvature of electron motion defined by the magnetic rigidity, *Bρ*[Tm] ≈ 3.33*p*[GeV/c], and azimuthal angle of electron motion, *θ*.

Describing the spiral motion of relativistic particles with transverse angle, *θ*, in this solenoidal field, *B*_*x*_, as 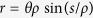 where *ρ* = *Bρ*/*B*_*x*_, yields the radius of curvature of this motion as 1/*R* = *d*^2^*r*/*ds*^2^ giving an estimate *R* = *Bρ*/(*B*_*x*_*θ*). The cooling length, *L*_*cool*_, can be defined to be equal to the distance over which the electron will lose all its transverse energy, estimated as *γm*_*e*_*c*^2^*θ*^2^, due to SR. Therefore its transverse emittance will decrease by a factor of *e* = 2.718 over *L*_*cool*_:


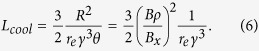


For a simple estimation 1 GeV electron energy, which means *γ* = 2000, and *B*_*x*_ = 10^5^ T magnetic field can be assumed resulting in *L*_*cool*_ ≈ 60*μ*m, which is much shorter than the pulse depletion length^34^: 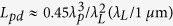

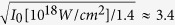
 mm in the case of Sim2. This type of effective cooling should improve overall beam emittance as beam will continue accelerate as shown in Fig. 8(e,f). The cooling can be less efficient when quantum effects start to dominate, i.e. the emitted photon energy becomes comparable to the electron energy. In the above example, the ratio *E*_*photon*_/*E*_*el*_ = *γ*^2^*r*_*e*_*B*/(*αBρ*) ≈ 0.05, where *α* ≈ 1/137, is much less then one but by increasing the field strength or the beam energy by one order of magnitude will get radiation close to offset of the quantum regime and estimations would need to be correspondingly adjusted. The longitudinal magnetic field will act not only to guide the electron beam (effective beam collimator) but also as an efficient coolant mediating conversion of beam transverse energy into high frequency synchrotron radiation at the same time.

### The steady solenoid

So far, only the bubble regime has been considered where laser spot size *W*_*L*_ < *λ*_*p*_ and spiral step *λ*_*sp*_ < *λ*_*p*_ were relatively small. In this case, the magnetic field moves with the speed of light and constantly appears and disappears in the plasma. This may be advantageous in some cases but this field is difficult to detect with direct methods. If the pulse length approaches or exceeds the bubble length, more uniform magnetic field can be observed (Figs 5b and 7a). In this regime, the azimuthal current can be resonantly driven if the plasma wavelength matches the spiral step of the laser pulse. However in this case, the bubble cannot be formed and as a result, the spiral currents are not limited to a short intervals of bubble shell thus surviving over a longer distance behind the laser pulse.

**Figure 9:**
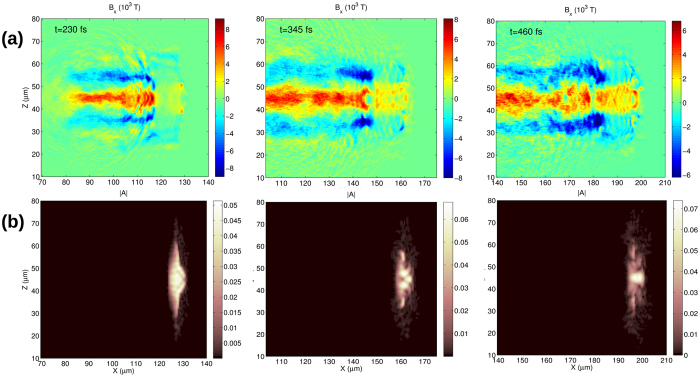
Longitudinal magnetic field **(a)** and amplitude of vector potential in the pulse **(b)** cross sections are shown at three time instances for the parameters of Sim5 with *I*_0_ = 0.8 × 10^21^W/cm^2^.

Figure 9 shows one example where the parameters are similar to Sim5 but lower intensity is used. This regime is observable already in Fig. 7a, because the parameter-set of Sim5 is close to the red area in Fig. 6, meaning a fast depletion (decreasing intensity) and transition to the yellow area. The energy distribution of the laser pulse is also shown in Fig. 9b, indicating energy depletion and strong modulations during the pulse evolution. It is clear that all interactions end with the transition from bubble to steady solenoid because of energy depletion. The positive and negative magnetic fields are parallel and separated in the channel behind the laser pulse, as expected in any solenoid structures. This structure brakes in the limit of *W*_*L*_ ≫ *λ*_*p*_, because of magnetic filaments evolving in the plasma if the current flow is wider than the local Debye-length.

The lifetime of magnetic fields generated in this way depends on the collision frequency between electrons and ions and the magnetic diffusion time, defined as *t*_*d*_ = *μ*_0_*R*^2^/*η*^35^, where *R* is the transverse scale length of the electron beam and *η* is the resistivity. The first effect is negligible in gases but if >kT magnetic fields are generated, the plasma density could be high enough to smear out the electron rotation. The second effect is also negligible because the plasma resistivity is on the order of 10^−6^ Ωm which gives more than 1 ps diffusion time with *R* ≈ 10 *μ*m. In the simulation shown in Fig. 9, the depletion time is on the order of 500 fs and the length of the quasi-static axial magnetic field can reach the sub-millimeter level which can be easily detected in experiments^16^.

## Conclusions and Outlook

We suggested and discussed the possibility of generating GigaGauss level axial magnetic field in underdense plasmas in laboratory environment using screw-shaped intense laser pulses. In this paper, the pulse length has been considered to be equal to the spiral period. In lower density plasmas (gas), the usual *λ*_*L*_ ≈ 1 *μ*m high power lasers (currently available to researchers) are suitable for reaching 10 kT fields co-moving with the laser pulse in a spatial volume comparable with the pulse length. It has been demonstrated that by changing the relative laser beam size with respect to the plasma wavelength, the shape of the axial magnetic field distribution can be tuned. If the ratio of *λ*_*p*_/*λ*_*sp*_ is near one, a static solenoid magnetic field is generated efficiently along a straight line behind the laser pulse limited only by the laser pulse energy depletion. If the plasma wavelength is relatively large, a bubble is formed with a strong, highly peaked and localized magnetic field at its tail. This regime becomes highly non-linear i.e. the introduced scaling does not work, and unstable if the bubble size, and the number of captured electrons, is too large.

We should note that in the presented paper we rely on the pulses of high intensity and of special shapes. Although the methods of generating such laser pulses, described, in particular, in ref. 26 are very preliminary and conceptual, one can be optimistic, as Atto-second laser science continues to demonstrate rapid progress and the necessary laser pulses can become feasible in very near future. The use of such a pulse could bring a numerous advantages to the fields of laser wake field acceleration or astrophysics. The observation of synchrotron radiation in standard PIC simulations requires very fine resolution, therefore extended codes are required capable of calculating sub-grid scale radiation. From the current results, it is anticipated that the spiral motion of well-bunched relativistic electron beam will result in intense synchrotron emission with small angular spread at nm wavelengths and estimates can be made of the corresponding parameters of synchrotron radiation and in particular of the cooling rate of the electron beam emittance.

Immersing electron bunches which are accelerated during laser plasma interaction into co-propagating axial magnetic field enables unique possibility to confine high density beam while improving beam emittance. We have demonstrated that large accelerating potential can be achieved simultaneously with strong longitudinal magnetic field at the point of beam self-injection. Showing that it is possible to use such accelerating structure will motivate future studies in this direction, in particular the investigation of transversal cooling of electrons due to synchrotron radiation. The non-uniform axial B-field can behave as a magnetic mirror allowing to observe such phenomena as beam reflection and trapping. The possibility of using screw-shaped relativistic electron beams instead of laser pulses is also one of the follow-up research topics triggered by this work.

## Additional Information

**How to cite this article**: Lécz, Z. *et al*. GigaGauss solenoidal magnetic field inside bubbles excited in under-dense plasma. *Sci. Rep.* **6**, 36139; doi: 10.1038/srep36139 (2016).

**Publisher’s note:** Springer Nature remains neutral with regard to jurisdictional claims in published maps and institutional affiliations.

## Supplementary Material

Supplementary Information

## Figures and Tables

**Table 1:** Simulation parameters.

Sim.	2*σ*_1_ (*μ*m)	*λ*_*sp*_ (*μ*m)	*λ*_*L*_ (nm)	*I*_0_ (W/cm^2^)	*εL* (J)	*n*_0_/*ncr*	max(*Bx*) (T)
1	1.8	0.9	100	1.6 × 10^21^	0.09	0.62 × 10^−3^	4000
2	1.2	0.9	100	8 × 10^21^	0.18	6.2 × 10^−3^	2.8 × 10^4^
3	0.8	0.9	100	3.2 × 10^22^	0.36	0.062	2.5 × 10^5^
4	0.3	0.3	20	8 × 10^23^	0.4	0.025	0.95 × 10^6^
5	14.4	7.2	800	1.6 × 10^21^	45	0.04	1.2 × 10^4^
6	14.4	7.2	800	0.8 × 10^21^	22.5	0.01	5000
7	14.4	7.2	800	3.2 × 10^21^	90	0.01	8000
8	9.6	7.2	800	2 × 10^22^	280	0.1	5 × 10^4^

σ_2_ = σ_1_/2 in all simulations and *ε*_*L*_ is the pulse energy.
