# Supplementary material for: GigaGauss solenoidal magnetic field inside bubbles excited in under-dense plasma
Source: Sci Rep. 2016 Oct 31;6:36139. doi: 10.1038/srep36139 (PMC5086957; doi:10.1038/srep36139)
Supplement: Supplementary Information [file srep36139-s1.pdf]

GigaGauss solenoidal magnetic field inside of bubbles excited in under-dense plasma

Zs. Lécz *[1], I. V. Konoplev[4], A. Seryi[4], and A. Andreev[1,2,3]

[1]ELI-ALPS, Dugonics Square 13, 6720 Szeged, Hungary
[2]Max-Born Institute, Berlin, Germany
[3] Sankt Petersburg State University, St. Petersburg, Russia
[4]John Adams Institute for Accelerator Science, and Physics Department, University of Oxford, Keble Road, Oxford OX1 3RH, UK

August 29, 2016

Implementation of the screw-shaped pulse

The cork-screw shape of the laser envelope can be obtained by implementing a rotating elliptic beam profile, with periodicity λ_{sp}. The transverse beam profile at one point of the pulse is the superposition of two Gaussian shapes: one is rotation symmetric with standard deviation σ_1 and one is uniform in the radial direction with $\sigma_2 < \sigma_1$ in the orthogonal direction. After some geometric calculations one can derive the function of a line rotating around the axis in the transverse plane, which will give the radial direction of the second Gaussian. Finally, the function describing the intensity distribution has the the following form:

$$I_L = I_0 \exp\left(-\frac{D^2}{2\sigma_2^2}\right) \exp\left(-\frac{r^2}{2\sigma_1^2}\right) \cos[(2x/L_{lp} - 1)\pi/2], \tag{1}$$

where L_{lp} is the total pulse length, $r = \sqrt{y^2 + z^2}$ and the distance from the rotating line in the transverse plane:

$$D(x, y, z) = [(y - \cos(\alpha)(z\tan(\alpha) + y))^2 + (z - \sin(\alpha)(z\tan(\alpha) + y))^2]^{1/2}, \tag{2}$$

where $\alpha = \pi x/\lambda_{sp}$ is the phase of the spiral envelope. In Eq. 1 a cosine function also appears, which is the longitudinal envelope. The usual Gauss function is not appropriate in this case, because it has to be truncated and the gradient at the leading edge of the pulse would be too large. By using this function we can assure that the intensity value is zero at both ends of the pulse.

The mathematical formulation presented above is suitable to define the pulse shape in numerical simulations, but it is not guaranteed that it can be realized in experiments as well. Here we propose a second method to generate pulses with helical intensity iso-surface. The pulse shape generated in the simulations is illustrated in Fig. 1. The cross sections along three vertical (in y direction) lines show that the wavefront is rotated around a diagonal axis (in z) of the pulse. In the middle the pulse is tightly focused and it is longer, because of the Gaussian envelope (see Eq. 1), and it can be produced if the wavefront rotation is applied to a wide Gaussian pulse. Such technique has been successfully used in experiment for the realization of attosecond lighthouse effect. The difference is that in our case the rotation should be performed along a radial direction not along the propagation axis of the pulse. With other words a special optical device is needed which imposes a helical phase delay on the transversal plane of the laser beam.

*Zsolt.Lecz@eli-alps.hu

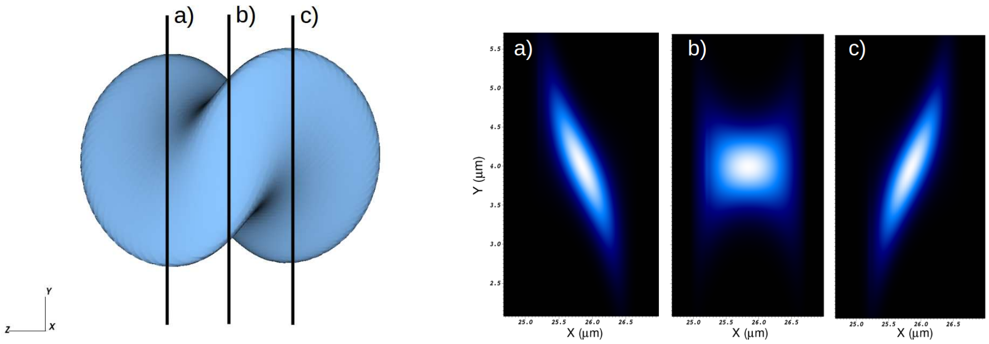

Figure 1: Left: iso-value surface of the pulse intensity generated in the simulation (example). Right: cross sections along the lines (a,b,c). The pulse width and length along (b) would be the same as in (a) and (c) in the case of wave-front rotation.

The simulation space contains 200 × 200 × 200 computational mesh cells with variable grid sizes depending on the bubble size. The propagation time is about 100 fs, which corresponded to about 1000 PIC cycles. The plasma is represented by electrons with under-critical density with 8 macro-particles in each cell. In order to simulate propagation distance much longer than the domain size we applied the moving window feature of the VSim code. In this case the simulation box is moving in the positive x direction with the speed of light and in each time step electrons are loaded in the new grid cell appearing at the front side and electrons are absorbed at the back side of the simulation domain.

Comparison with full EM solver

The last simulation (Sim8) has been repeated using real laser pulse instead of defining the intensity envelope only. In this case the grid resolution is much higher, $dx = 20$ nm, which is required to resolve the laser wavelength. This simulation took 30 times longer than the one performed with the envelope model. The direct comparison of the generated magnetic field is shown in Fig. 2, which reveals some discrepancies in the spatial structure, but the absolute values are the same in both cases. The detailed field distribution can not be reproduced in the envelope model simulations due to the large grid size. The small scale variations can be attributed to the wave representation of the laser field, which inevitably results in oscillating ponderomotive force, or to diffraction effects appearing near the edge of the transversal intensity profile.

The numerical precision and fidelity of the envelope model can be further proven by comparing the time evolution of electron energy gained during interaction. The total energy of electrons measured at each time step is presented in Fig. 3 for both simulations. The agreement is very good, which shows that the envelope model can be used for simulation of laser-plasma interaction, at least in the underdense regime.

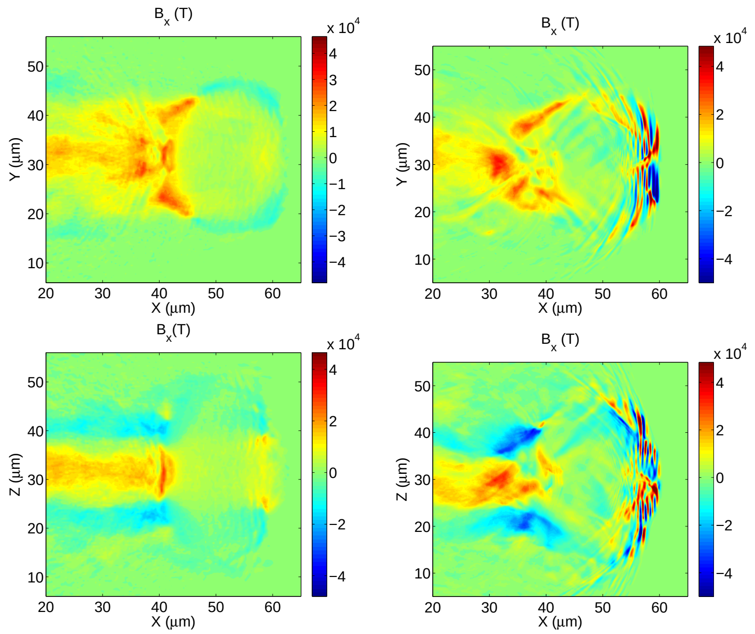

Figure 2: Longitudinal magnetic field in simulation 8 with envelope (left) and real (right) pulse.

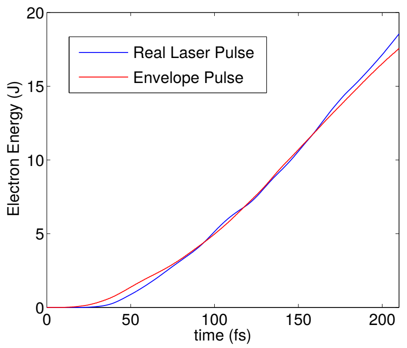

Figure 3: Total energy of electrons inside the simulation box.
